# T cell immune senescence is associated with frailty and sarcopenia in lung transplant candidates

**DOI:** 10.1016/j.jhlto.2024.100199

**Published:** 2024-12-20

**Authors:** Joanna M. Schaenman, Harry Pickering, Elaine F. Reed, Maura Rossetti, Benjamin Seligman, S. Samuel Weigt, Michael Shino, David Sayah, John Belperio, Ashley Hu, Ashley Prosper, Kathleen Ruchalski, Abbas Ardehali, Reshma Biniwale

**Affiliations:** a Department of Medicine, Division of Infectious Diseases, David Geffen School of Medicine, Los Angeles, California; b Department of Pathology and Laboratory Medicine, David Geffen School of Medicine, Los Angeles, California; c Department of Medicine, Division of Geriatrics, David Geffen School of Medicine, Los Angeles, California; d Department of Medicine, Division of Pulmonary Medicine, David Geffen School of Medicine, Los Angeles, California; e Department of Radiology, David Geffen School of Medicine, Los Angeles, California; f Department of Cardiothoracic Surgery, David Geffen School of Medicine, Los Angeles, California

**Keywords:** T cell, immune senescence, frailty, sarcopenia, lung transplantation, outcomes

## Abstract

**Backgound:**

Older lung transplant recipients experience increased rates of adverse clinical outcomes, including infection compared with younger patients, potentially related to impaired cell-mediated immunity, frailty, and sarcopenia.

**Methods:**

Patients over age 55 years undergoing evaluation for lung transplantation were evaluated for sarcopenia by cross-sectional area and average attenuation of the pectoralis major muscle on chest computed tomography. Frailty was measured using the Fried Frailty Phenotype. Immune phenotyping was performed using multichannel flow cytometry of peripheral blood mononuclear cells (PBMC) in a total of 26 lung transplant candidates.

**Results:**

The median patient age was 65, primarily with restrictive lung disease (76.9%). Hospital readmission was associated with lower frequency of naïve CD4 (*p* = 0.004) and CD8 T cells (*p* = 0.026). Senescent CD4 (KLRG1+/CD28−) and CD8 T cells were also associated with readmission (*p* = 0.014 and *p* = 0.013, respectively), and senescent CD4 T cells were predictive of total hospital time (*p* = 0.003). TEMRA CD4 T cells were significantly associated with frailty (*p* = 0.015) and sarcopenia (*p* = 0.011). Senescent CD4 and CD8 T cells were significantly associated with sarcopenia (*p* = 0.009 and *p* = 0.006, respectively).

**Conclusions:**

These findings suggest that impaired cell-mediated immunity may underlie the associations between frailty and sarcopenia and poor clinical outcomes. A multifaceted approach to evaluation of older patients has the potential to improve risk stratification and inform management of immunosuppression.

## Background

Older lung transplant (LT) recipients experience increased adverse clinical outcomes, including infection after transplantation compared with younger patients, potentially related to impaired cell-mediated immunity.^1^ Assessment of frailty and sarcopenia has been shown to aid in patient risk stratification in transplant candidates. However, little is known about their association with age–associated T cell dysfunction.

In 2021, 34.3% of LT candidates were 65 years or older, and those 65 years or older increased in number by 67.0% compared with 2010.^2^ However, these older patients experience increased mortality on the waitlist compared with younger patients, with mortality rates nearly 2-fold of those aged 35 to 64 and more than 4-fold of candidates aged 18 to 34 years. In addition, older LT recipients have significantly lower patient survival compared with younger patients.^2^ One driver of the increased age of LT candidates and recipients are increased incidence of idiopathic pulmonary fibrosis, a disease that is strongly associated with patient age and has been shown to be driven, at least in part, by cellular senescence.^3^, ^4^, ^5^

A growing body of literature has developed regarding the cellular senescence-related onset of sarcopenia, defined as decreased muscle quality and/or quantity.^6^ Sarcopenia as measured by computed tomography (CT) evaluation of the chest has been shown by our group and other authors as associated with mortality and length of stay (LOS) and ventilation days after transplantation.^7^, ^8^, ^9^, 10. Various anatomic locations have been utilized for assessment, including the carina, thoracic, and lumbar vertebrae. Frailty measurements, including frailty index, Fried Frailty Phenotype (FFP), or Short Performance Physical Battery (SPPB), have also been shown to be associated with days of hospitalization.^11^, ^12^, ^13^, ^14^

Immune aging is associated with increased frequency of senescent and terminally differentiated T cells, characterized by impaired proliferation in response to antigen stimulation, and decreased frequency of naïve T cells, leading to increased vulnerability to infection and death in older patients.^15^ Senescent T cells exhibit impaired ability to proliferate in response to antigen stimulation, and they are often characterized by loss of CD28 as well as by expression of the killer cell lectin-like receptor G1 (KLRG1) inhibitory surface molecule.^16^, ^17^ However, little is known about the association between T cell senescence and outcomes after LT. In addition, although there is published data connecting measures of inflammation with frailty or sarcopenia and impaired outcomes after transplantation,^18^ there is a gap of knowledge in terms of T cell phenotype and frailty or sarcopenia in transplant recipients. Given the known association between inflammation or “inflammaging” via the senescence–associated secretory phenotype, it is to be expected that T cell senescence would play an important role in the mechanism of age-associated impairment, such as sarcopenia.

We therefore sought to evaluate T cell phenotype, sarcopenia, and frailty in LT candidates with end-stage lung disease to determine whether there is in fact interaction between these factors as well as whether incorporation of T cell assessment could improve prediction of clinical outcomes after transplantation. We therefore utilized a patient cohort at our center where frailty, sarcopenia, and T cell immune phenotyping data were available.

## Methods

Patients over age 55 years undergoing evaluation for LT were enrolled. Sarcopenia was measured using cross-sectional area and average attenuation of the pectoralis major muscle at 1 slice above the aortic arch on pretransplant chest CT. Frailty was measured using the FFP and SPPB as previously described.^10^ Immune phenotyping was performed using multichannel flow cytometry at the time of transplant evaluation as previously described, including assessment of CD3, CD4, CD8, CCR7, CD45RA, KLRG1, CD57, CD38, CD28, and PD-1 (BD Biosciences or BioLegend)^19^ (Supplementary Figure).

As described previously, this study was approved by the University of California, Los Angeles (UCLA) IRB. Patients aged 55 and older undergoing evaluation for LT at our center were enrolled and underwent testing of FFP and SPPB during outpatient clinic visits. Sarcopenia was evaluated through measurement of cross-sectional area (CSA) and average of attenuation of bilateral pectoralis major muscles 1 slice above the aortic arch on CT chest obtained as part of the standard clinical assessment; OsiriX software was used as described previously.^10^ The electronic medical record was reviewed to determine patient demographic characteristics, cause of lung disease, death, transplantation, LOS after LT, hospital readmission, and total hospital time in the first year after transplantation.

Statistical analysis was performed with the JMP Pro 17 (SAS software). Nonparametric analysis was performed for numeric variables using the Wilcoxon/Kruskal-Wallis test and Pearson chi-square test was used to determine the association between 2 categorical variables. Linear regression was used to compare numeric values. A *p*-value <0.05 was considered statistically significant.

## Results

### Patient cohort characteristics and comparison with larger cohort

Of the 84 patients with CT scans and frailty measurements, this study focused on 26 patients who had T cell phenotyping data available. Median patient age was 65, primarily with restrictive lung disease including idiopathic pulmonary fibrosis (76.9%) (Table 1). The majority of the cohort was male sex with median LAS of 38. All patients underwent transplantation, with a median of 1 readmission after the initial transplant admission (range 0-3). The median hospital LOS after transplantation was 14 days, and the median total time in hospital in the first year after transplantation was 19 days (Table 2). These characteristics were similar to the larger cohort from which study patients were derived.^10^

**Table 1:** Demographic Characteristics of Patients Studied

Demographic characteristic	*n* = 26
Patient age	65 (60-72)
Female sex	38.5%
Race/ethnicity	
Non-Hispanic white	73.1%
Hispanic	15.4%
Other	11.5%
Diagnosis group	
A	19.2%
B	3.8%
C	0.0%
D	76.9%
LAS score	38 (28-78)
BMI	25.5 (15.5-32.0)

Abbreviations: LAS, lung allocation score; BMI, body mass index.

Values are median followed by range. Group A = obstructive lung disease (e.g., emphysema); group B = pulmonary vascular disease (e.g., primary pulmonary hypertension); group C = cystic fibrosis or immunodeficiency disorder; group D = restrictive lung disease (e.g., idiopathic pulmonary fibrosis).

**Table 2:** Frailty and Sarcopenia Measures in Patient Cohort

Clinical outcomes	*n* = 26
Transplanted	100.0%
Single lung transplant	61.5%
LOS	14 (8-177)
Readmission first year	72.0%
No. of readmissions	1 (0-3)
Total time in hospital first year	19 (8-276)
Biopsy-proven rejection	30.8%
Death post transplant	26.9%

Abbreviation: LOS, length of stay.

Values are median followed by range.

### Immune phenotypes are associated with negative outcomes

**Figure 1:**
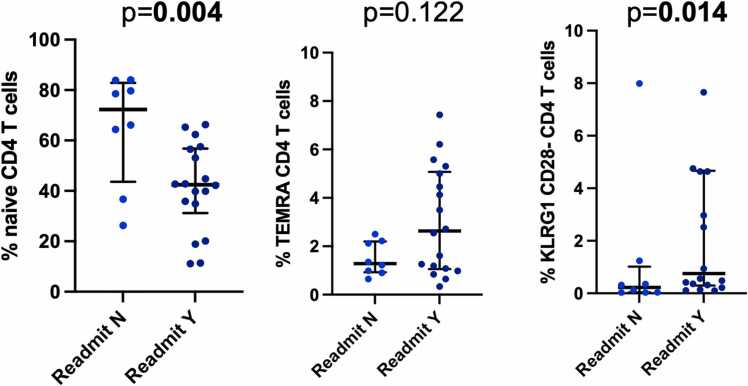
Dot plot showing the median and interquartile range of the frequency of the cell type indicated by readmission status in the first year after lung transplantation. Cell types depicted are CD4 naïve, terminally differentiated RA+ (TEMRA), and senescent KLRG1/CD28− T cells. Bold indicates *p* < 0.05. KLRG1, killer cell lectin-like receptor G1.

**Figure 2:**
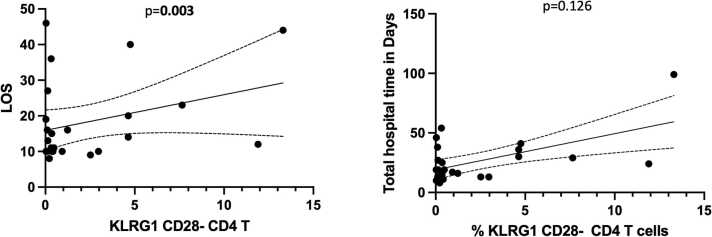
Linear regression demonstrating the relationship between senescent KLRG1/CD28− CD4 T cells and length of stay (LOS) (left panel) and total hospital time in days (right panel). Bold indicates *p* < 0.05. KLRG1, killer cell lectin-like receptor G1.

We evaluated the association between immunologic phenotypes of maturation and senescence and clinical outcomes after transplantation. We found that readmission after the initial transplant hospitalization was significantly associated with lower frequency of CD4 naïve T cells (*p* = 0.004) and naïve CD8 T cells (*p* = 0.026). The median frequency of CD4 naive T cells was 42.2% in patients who required readmission compared with 78.5% in those who did not require readmission (*p* = 0.004) (Figure 1). For CD8 naive T cells, frequency was 25.2% for patients who required readmission compared with 51.1% in patients who did not require readmission (*p* = 0.026). The overall frequency of central memory (CM), effector memory (EM), and terminally differentiated RA+ T cells (TEMRA) was not significantly associated with readmission, with the exception of CD4 CM T cells, which were higher in patients who required readmission (30.6%) compared with those who did not (17.4%) (*p* = 0.033) (Table 3). Senescent CD4 (KLRG1+/CD28-) and CD8 T cells were also associated with readmission (*p* = 0.014 and *p* = 0.013, respectively). The median frequency of CD4 senescent T cells was 0.94% in patients who required readmission compared with 0.13% in those who did not require readmission (Figure 1); the median frequency of CD8 senescent T cells was 4.3% in those who required readmission compared with 2.7% in those who did not require readmission. T cell phenotype was not significantly associated with LOS after transplantation; however, analysis by total hospital time during the first year post transplantation demonstrated a significant association with senescent CD4 T cells with total hospital time (*p* = 0.003) (Figure 2). Other T cell phenotypes were not significantly associated with total hospital time (Supplementary Table).

**Table 3:** T Cell Phenotypes by Readmission Status

T cell attributes	Readmission No		Readmission Yes		
	Median %	IQR	Median %	IQR	*p*-value
CD4 naïve	78.5	64.4-83.9	42.5	31.2-56.8	**0.004**
CD4 CM	17.4	9.1-27.3	30.1	21.2-40.8	**0.032**
CD4 EM	6.0	4.4-12.0	23.2	13.5-29.4	**0.002**
CD4 TEMRA	1.2	0.9-2.1	2.6	1.1-5.1	0.122
CD4 KLRG1+/CD28−	0.1	0.04-0.35	0.8	0.3-4.7	**0.014**
CD8 naïve	51.1	36.9-90.4	24.7	11.8-47.9	**0.020**
CD8 CM	4.4	0.5-10.2	5.9	3.4-10.7	0.545
CD8 EM	14.2	1.2-19.3	19.1	13.1-28.9	0.138
CD8 TEMRA	24.6	8.1-26.4	40.0	17.1-56.5	0.074
CD8 KLRG1+/CD28−	2.7	0.2-3.2	4.7	2.8-16.0	**0.010**

Abbreviations: CM, central memory; EM, effector memory; IQR, interquartile range; KLRG1, killer cell lectin-like receptor G1; TEMRA, terminally differentiated RA+ T cells.

Values are median and IQR. Bold indicates *p < 0.05.*

### Immune phenotypes are associated with frailty

**Figure 3:**
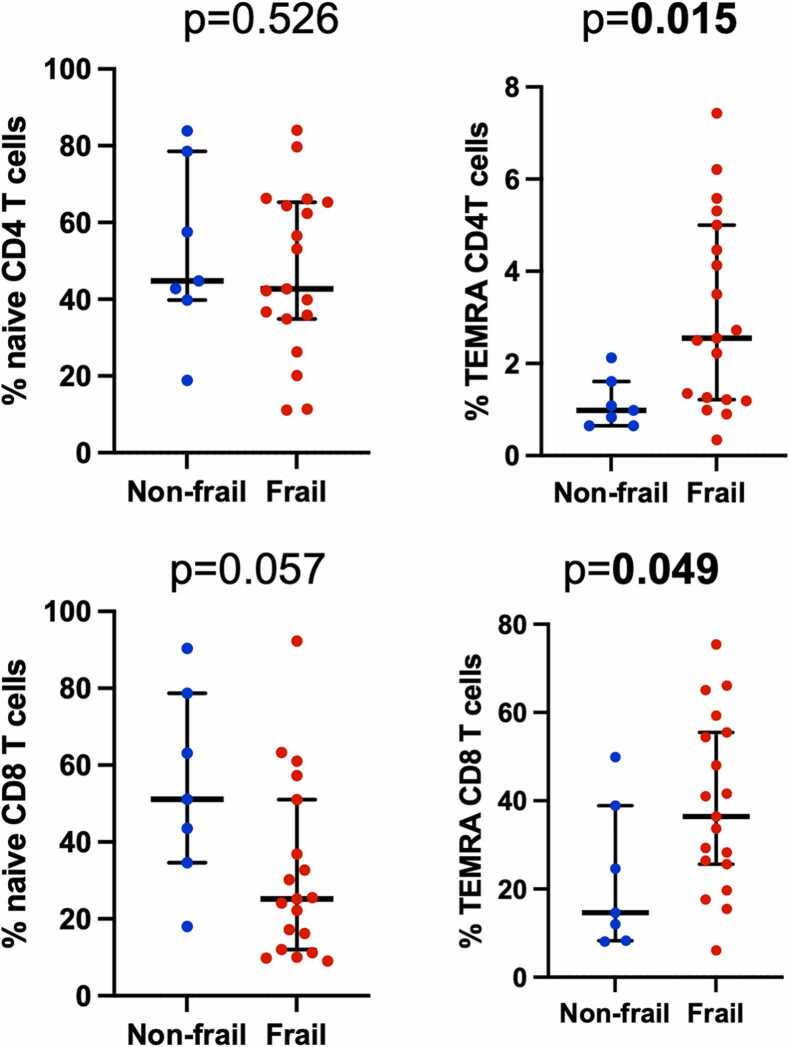
Dot plot showing the median and interquartile range of the frequency of the cell type indicated by frailty status by Fried Frailty Phenotype measured before lung transplantation. Nonfrail category includes patients designated nonfrail and prefrail. Cell types depicted are CD4 naïve and terminally differentiated RA+ (TEMRA) T cells. Bold indicates *p* < 0.05.

Frailty was assessed by FFP and SPPB at the time of evaluation clinic visit. We found that overall percentage of CD4 and CD8 T cells were associated with frailty as measured by SPPB, with 84.2% CD4 T cells observed in prefail or frail patients compared with 66.0% in nonfrail patients (*p* = 0.049), and that prefrail or frail patients demonstrated 12.6% CD8 T cells compared with 30.0% in nonfrail patients (*p* = 0.049). However, CD4 or CD8 T cell subtypes were not significantly associated with frailty by SPPB. Analysis of frailty by FFP, in contrast, demonstrated that TEMRA CD4 T cells were significantly associated with frailty by FFP, with 3.1% frequency in frail patients compared with 0.98% in nonfrail patients (*p* = 0.015) (Figure 3; Table 4). Naïve, CM, EM, or senescent CD4 T cells were not associated with frailty by FFP. TEMRA CD8 T cells also demonstrated an association with frailty as measured by FFP, 39.0% frequency in frail compared with 14.6% in nonfrail patients (*p* = 0.049) (Figure 3). Naïve, CM, EM, or senescent CD4 T cells were not associated with frailty by FFP.

**Table 4:** T Cell Phenotypes by Frailty Status by FFP

T cell attributes	Frail		Nonfrail/prefrail		
	Median %	IQR	Median %	IQR	*p*-value
CD4 naïve	42.5	35.2-65.1	44.8	39.8-78.5	0.526
CD4 CM	26.3	19.2-36.2	32.4	17.4-45.4	0.404
CD4 EM	22.0	12.3-28.5	13.6	6.9-23.2	0.216
CD4 TEMRA	3.1	1.2-5.2	0.98	0.65-1.6	**0.015**
CD4 KLRG1+/CD28−	1.1	0.13-4.6	0.35	0.10-0.42	0.300
CD8 naïve	25.4	11.4-52.2	51.1	18.0-78.7	0.057
CD8 CM	4.8	1.9-9.8	6.2	4.1-10.7	0.713
CD8 EM	20.3	13.6-29.0	11.6	6.8-14.2	0.116
CD8 TEMRA	39.0	21.2-58.4	14.6	8.3-38.9	**0.049**
CD8 KLRG1+/CD28−	4.1	2.5-12.3	3.3	0.66-3.5	0.367

Abbreviations: CM, central memory; EM, effector memory; FFP, Fried Frailty Phenotype; IQR, interquartile range; KLRG1, killer cell lectin-like receptor G1; TEMRA, terminally differentiated T cells.

Values are median and IQR. Bold indicates *p < 0.05.*

To evaluate whether these associations were related to chronologic age and not solely to frailty, we evaluated for association between age and T cell phenotypes. This evaluation demonstrated no significant association between age and maturation phenotype or senescence: we found that neither naïve, CM, EM, TEMRA, nor senescent CD8 T cells were associated with patient age in this cohort of older patients with advanced lung disease (*p* > 0.05 for all subtypes). Similarly, neither naïve, CMV, TEMRA, nor senescence CD4 T cells were associated with patient age (*p* > 0.05 for all subtypes).

### Immune phenotypes are associated with sarcopenia

**Figure 4:**
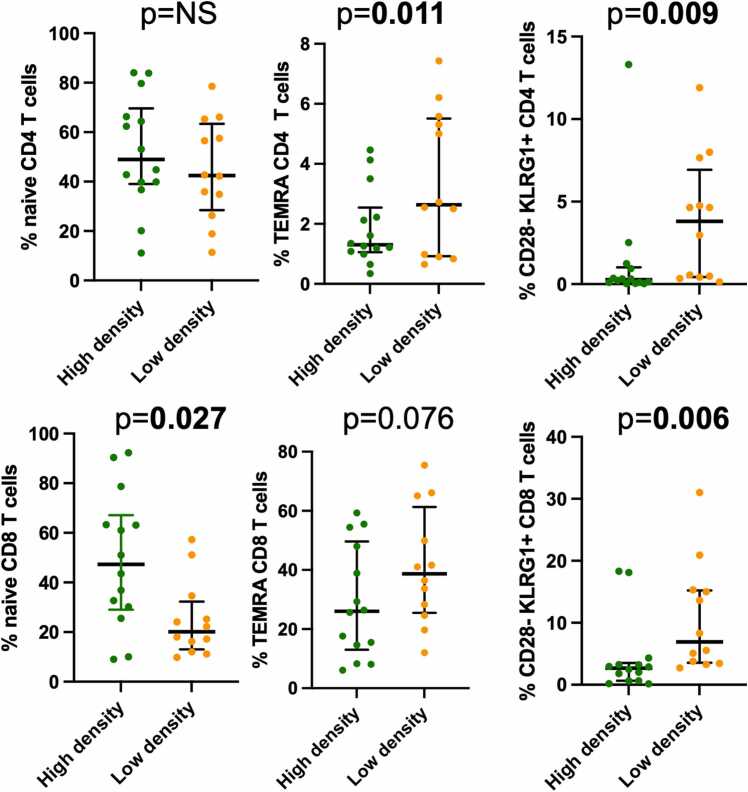
Dot plot showing the median and interquartile range of the frequency of the cell type indicated by sarcopenia status by attenuation measured before lung transplantation. High-density vs low-density categories are defined by median attenuation by sex. Cell types depicted are CD4 naïve and terminally differentiated RA+ (TEMRA) T cells and senescent KLRG1/CD28− T cells. Bold indicates *p* < 0.05. KLRG1, killer cell lectin-like receptor G1.

Sarcopenia was measured by total muscle area (quantity) as well as muscle attenuation (quality), as previously described. No significant association was seen between naïve CD4 or CD8 T cells and sarcopenia. In contrast, TEMRA CD4 T cells were significantly associated with sarcopenia by muscle attenuation by linear regression (*p* = 0.011) (Figure 4). In addition, categorical analysis of high vs low muscle attenuation by sex also demonstrated a trend toward association between sarcopenia and TEMRA CD4 T cells (*p* = 0.076). In contrast, TEMRA CD8 T cells were not significantly associated with sarcopenia by muscle attenuation, but frequency of naïve CD8 T cells was significantly associated with a frequency of 47.3% for those nonsarcopenic patients with high muscle attenuation compared with 18.0% for sarcopenic patients with low attenuation (*p* = 0.027). Evaluation was also performed for association with senescent T cells. Senescent CD4 and CD8 T cells were significantly associated with high vs low muscle attenuation (*p* = 0.009 and *p* = 0.006, respectively) (Figure 4). Sarcopenic patients with low muscle attenuation demonstrated 4.6% frequency of senescent CD4 T cells compared with 0.27% for nonsarcopenic patients, while senescent CD8 T cells demonstrated a frequency of 5.5% in sarcopenic patients compared with 2.7% in nonsarcopenic patients.

**Figure 5:**
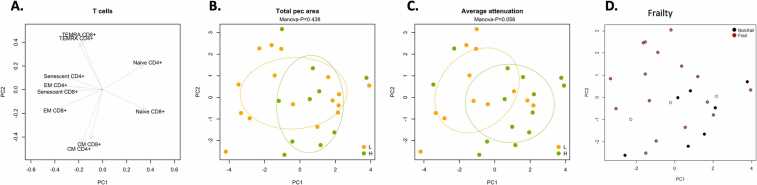
Principal component analysis (PCA) of T cell phenotypes analyzed demonstrating association with sarcopenia and frailty. Panel (A) demonstrates the 2 dominant PCA groups identified, PC1 and PC2. Panel (B) demonstrates separation by PCA analysis by total pectoral muscle area, with high area in green (H), and low area indicated in orange (L). Panel (C) demonstrates separation by PCA analysis by average attenuation, with high attenuation in green (H), and low attenuation indicated in orange (L). Panel (D) demonstrates separation by PCA analysis by frailty status by Fried Frailty Phenotype, with nonfrail (nonfrail or prefrail) patients in black and frail patients in red. KLRG1, killer cell lectin-like receptor G1.

Principal component analysis (PCA) of T cell phenotypes demonstrated 2 important PCA factors encompassing naïve and CM CD4 and CD8 T cells (PC1) compared with senescent, EM, and TEMRA CD4 and CD8 T cells (PC2) (Figure 5A). Using this approach, we were able to define patients with either low and high muscle area (Figure 5B) and low and high muscle attenuation (Figure 5C). This correlation further demonstrates the strong correlation between T cell phenotypes and sarcopenia in this cohort of LT candidates.

## Discussion

Age–related T cell immune phenotypes are associated with outcomes after lung transplantation in older patients. The associations of frailty and sarcopenia with T cell terminal differentiation and senescence suggest that impaired cell-mediated immunity may underlie their associations with poor clinical outcomes. A multifaceted approach to the evaluation of older patients can improve risk stratification, leading to individualization of immune suppression. Although previous authors have reported associations between inflammation cytokines and frailty or sarcopenia, this report represents the first demonstration of the association between T cell maturation phenotype and senescence with frailty and sarcopenia. In addition, we uniquely demonstrate an association between decreased naïve and increased senescent T cell subtypes with the clinically relevant outcomes of hospital readmission and total hospital time in the first year after transplantation. By combining cellular, frailty, and sarcopenia-based evaluation of transplant candidates, we will be able to create more complete compositive assessment of patients, especially for older patients at risk for poor outcomes with immunosuppression.

Many published studies in the field of lung transplantation have demonstrated the ability of frailty assessment to predict outcomes after transplantation with more fidelity than chronologic age.^1^, ^14^, ^20^ Our previous publication on sarcopenia demonstrated a significant association between successful transplantation as well as LOS after lung transplantation.^10^ Importantly, these measures of frailty or sarcopenia predict adverse outcomes before or after transplantation regardless of patient chronologic age. Our previous studies demonstrated that immunologic analysis of PBMC such as DNA methylation age is more closely associated with developmental infection compared with chronologic age.^21^ Therefore, the concept of improving risk stratification by adding immune system measures to frailty studies, such as markers of inflammation or senescence, has been advocated by many in the field given the promise of this type of multilevel assessment. Given the concept of frailty as measuring biologic age, it is reasonable to measure T cell maturation and senescence to determine whether this immune assessment is predictive of outcomes and associated with frailty and sarcopenia. The link between senescence–associated secretory phenotype and other senescent T cells with inflammation and acceleration of biologic age through common mechanism.

Although many authors have studied CD8 T cells in the context of aging, we observed in our study a more striking relationship between CD4 T cell maturation subtypes as a predictor of clinical outcomes and as associated with both frailty and sarcopenia. This may indicate the central role of CD4 T cells in coordinating the immune response including levels of inflammation. Studies from mouse models have clearly demonstrated that landscape of CD4 T cell subsets differs significantly in the old compared with the young.^22^ The loss of the naïve CD4 T cell population impairs ability to respond to novel antigens and may explain the association between this population and hospital readmission. In addition, accumulation of senescent T cells is also associated with impaired immune response and low-grade chronic inflammation. We found an association with senescent CD4 T cells and hospital readmission as well as total hospital time, as well as a striking association with sarcopenia as measured by muscle attenuation. This association with sarcopenia, with decreased muscle attenuation seen in patients with increased frequency of senescent cells, was observed for both CD4 and CD8 T cells. Future studies will evaluate functional aspects of T cell senescence such as proliferation and antigen-specific response to confirm the association with frailty and sarcopenia by T cell immune phenotyping performed for this study.

There have been previous publications on the relationship between frailty and inflammatory cytokines, but again, there are no previous reports on T cell associations to our knowledge. Interestingly, we noted the association between frailty by FFP but not SPPB and terminally differentiated CD4 and CD8 T cells. This observation may be explained by the fact that FFP which uses 5 distinct assessments to measure frailty, including exhaustion, shrinking, and weakness, is more of a systemic measure compared with SPPB, which focuses on lower extremity strength, so that FFP is more likely to be associated systemic immunologic differences.

The development of a combined testing modality that utilizes parallel assessment of age-associated factors, including T cell phenotype, would be a significant addition to candidate evaluation and patient risk stratification. In addition, this pretransplant immune system could be incorporated into decision-making for choices in induction and maintenance regimens, allowing for individualization of immunosuppression to avoid rejection while decreasing overimmunosuppression and its associated risks for infection and malignancy.

The primary limitation of this reported analysis is the relatively small cohort size. Future investigations can combine T cell assessment with cytokine-based measurement of inflammation to extend and validate these findings in a larger patient cohort.

## Author contributions

Joanna Schaenman: Conceptualization, Writing—Original draft preparation, Supervision, Funding acquisition. Harry Pickering: Methodology, Formal analysis, Visualization, Writing—Reviewing and Editing. Elaine Reed: Writing—Reviewing and Editing. Maura Rossetti: Methodology, Investigation. Benjamin Seligman, Samuel Weigt, Michael Shino, David Sayah, John Belperio: Conceptualization, Writing—Reviewing and Editing. Ashley Hu, Ashley Prosper, Kathleen Ruchalski: Methodology, Validation, Investigation. Abbas Ardehali: Writing—Reviewing and Editing. Reshma Biniwale: Conceptualization, Supervision, Writing—Reviewing and Editing.

## Disclosure statement

The authors have no relevant conflicts of interest to disclose.

We thank the research volunteers Christian Fulinara and Alina Huynh and others who assisted with the implementation of this project. Funding was provided in part by the National Center for Advancing Translational Sciences of the National Institutes of Health.

## Data Availability

The data that support the findings of this study are available on request from the corresponding author (J.S.). The data are not publicly available due to their containing information that could compromise the privacy of research participants.

## Appendix A Supplementary data

.

Supplementary Figure: Gating strategy and representative dot plots for flow cytometry analysis. After gating by forward scatter (FSC) and side scatter (SSC) and selection using a live/dead stain, CD3 expressing cells were gated by CD4 or CD8 followed by CD45RA and CCR7 to define maturation status and CD28 and KLRG1 to define senescent T cells
