# Supplementary material for: T cell immune senescence is associated with frailty and sarcopenia in lung transplant candidates
Source: JHLT Open. 2024 Dec 20;7:100199. doi: 10.1016/j.jhlto.2024.100199 (PMC11935382; doi:10.1016/j.jhlto.2024.100199)
Supplement: Supplementary file 1 — Supplementary material [file mmc1.docx]

**Supp. Table**: T cell phenotypes by total hospital time. Bold indicates p<0.05.

| **T cell attributes by total hospital time** | **Rsquare** | **p-value** |
| --- | --- | --- |
| CD4 naïve | 0.001 | 0.891 |
| CD4 CM | 0.007 | 0.702 |
| CD4 EM | 0.007 | 0.670 |
| CD4 TEMRA | 0.087 | 0.162 |
| CD4 KLRG1+/CD28- | 0.329 | **0.003** |
| CD8 naïve | 0.038 | 0.361 |
| CD8 CM | 0.017 | 0.546 |
| CD8 EM | 0.004 | 0.756 |
| CD8 TEMRA | 0.052 | 0.284 |
| CD8 KLRG1+/CD28- | 0.031 | 0.408 |
