# Supplementary figures and images for: T cell immune senescence is associated with frailty and sarcopenia in lung transplant candidates
Source: JHLT Open. 2024 Dec 20;7:100199. doi: 10.1016/j.jhlto.2024.100199 (PMC11935382; doi:10.1016/j.jhlto.2024.100199)

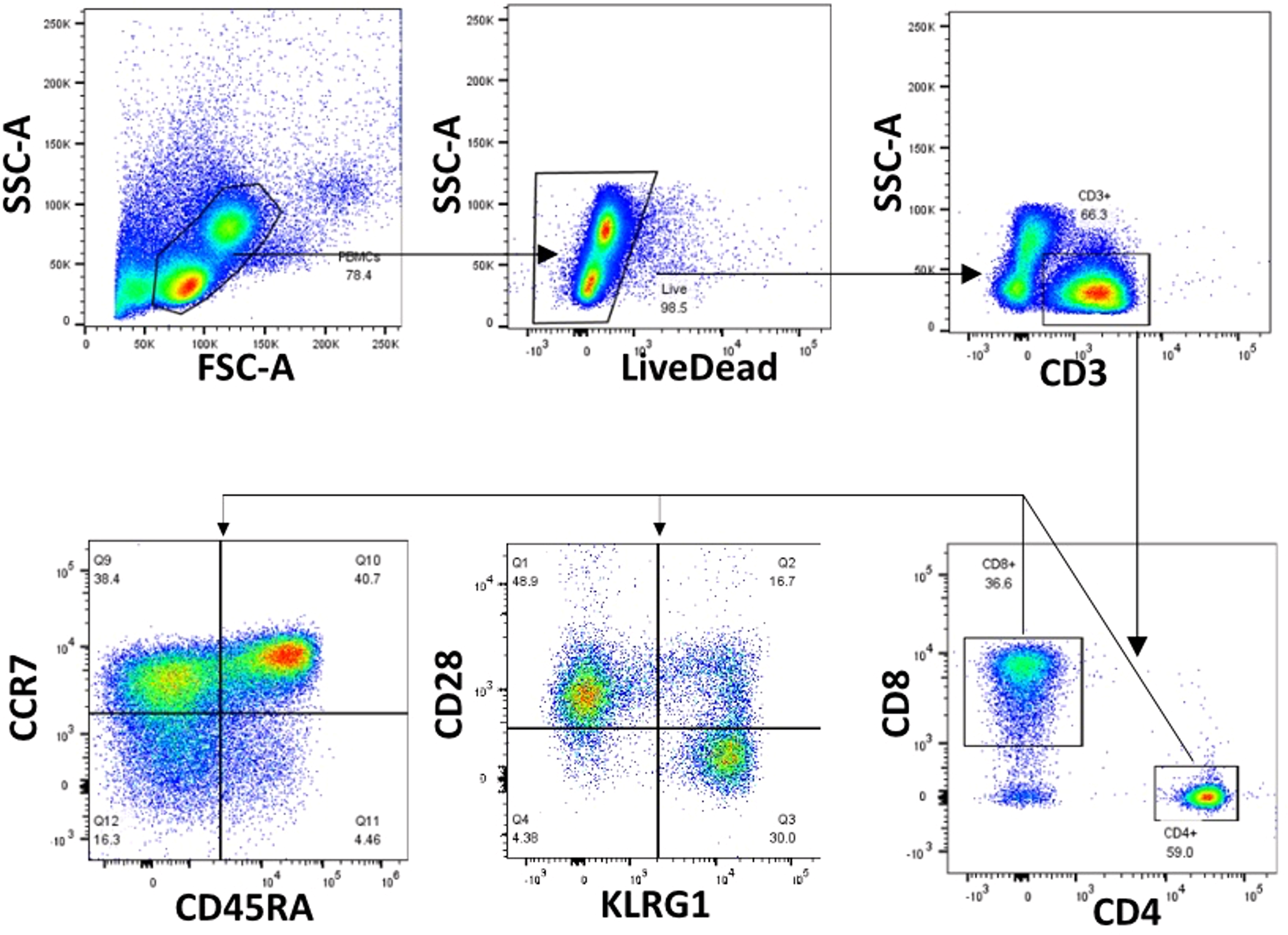

Supplement: Supplementary file 2 — Supplementary Figure: Gating strategy and representative dot plots for flow cytometry analysis. After gating by forward scatter (FSC) and side scatter (SSC) and selection using a live/dead stain, CD3 expressing cells were gated by CD4 or CD8 followed by CD45RA and CCR7 to define maturation status and CD28 and KLRG1 to define senescent T cells [file mmc2.jpg]
